# Mass Cytometry Phenotyping of Human Granulocytes Reveals Novel Basophil Functional Heterogeneity

**DOI:** 10.1016/j.isci.2020.101724

**Published:** 2020-10-22

**Authors:** Nora Vivanco Gonzalez, John-Paul Oliveria, Dmitry Tebaykin, Geoffrey T. Ivison, Kaori Mukai, Mindy M. Tsai, Luciene Borges, Kari C. Nadeau, Stephen J. Galli, Albert G. Tsai, Sean C. Bendall

**Affiliations:** 1 Department of Pathology, School of Medicine, Stanford University, Stanford Blood Center, 3373 Hillview Avenue Room 230A, Palo Alto, CA 94305, USA; 2 Department of Medicine, Division of Respirology, McMaster University, Hamilton, ON, L8S4K1, Canada; 3 Sean N. Parker Center for Allergy Research, School of Medicine, Stanford University, Palo Alto, CA 94305, USA; 4 Department of Microbiology and Immunology, School of Medicine, Stanford University, Palo Alto, CA 94305, USA; 5 Department of Medicine, Division of Pulmonary and Critical Care Medicine, School of Medicine, Stanford University, Palo Alto, CA 94305, USA

**Keywords:** Immunology, Systems Biology

## Abstract

Basophils, the rarest granulocyte, play critical roles in parasite- and allergen-induced inflammation. We applied mass cytometry (CyTOF) to simultaneously asses 44 proteins to phenotype and functionally characterize neutrophils, eosinophils, and basophils from 19 healthy donors. There was minimal heterogeneity seen in eosinophils and neutrophils, but data-driven analyses revealed four unique subpopulations within phenotypically basophilic granulocytes (PBG; CD45^+^HLA-DR^−^CD123^+^). Through CyTOF and fluorescence-activated cell sorting (FACS), we classified these four PBG subpopulations as (I) CD16^low^FcεRI^high^CD244^high^ (88.5 ± 1.2%), (II) CD16^high^FcεRI^high^CD244^high^ (9.1 ± 0.4%), (III) CD16^low^FcεRI^low^CD244^low^ (2.3 ± 1.3), and (IV) CD16^high^FcεRI^low^CD244^low^ (0.4 ± 0.1%). Prospective isolation confirmed basophilic-morphology of PBG I–III, but neutrophilic-morphology of PBG IV. Functional interrogation via IgE-crosslinking or IL-3 stimulation demonstrated that PBG I–II had significant increases in CD203c expression, whereas PBG III–IV remained unchanged compared with media-alone conditions. Thus, PBG III–IV could serve roles in non-IgE-mediated immunity. Our findings offer new perspectives in human basophil heterogeneity and the varying functional potential of these new subsets in health and disease.

## Highlights

• Unsupervised clustering revealed 4 basophil populations, driven by CD16, CD244, and FcεRI
• The rarest basophil subpopulation IV was morphologically neutrophils
• Anti-IgE and IL-3 stimulation did not induce functional responses in III and IV
• Basophil subpopulation heterogeneity was observed in healthy and CML samples

## Graphical Abstract

**Figure:**
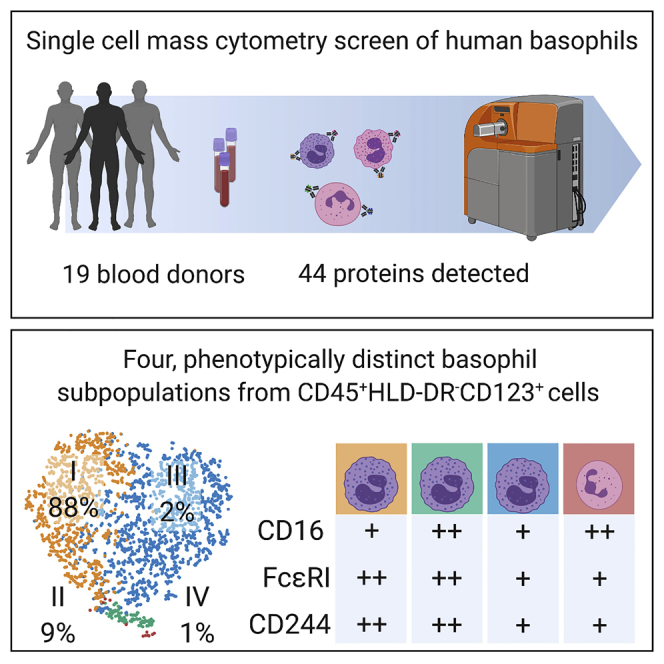

## Introduction

Granulocytes, including eosinophils, neutrophils, and basophils, have diverse and critical roles in disease propagation, including clearing pathogens and initiating inflammatory processes in cancer (Carruale et al., 2019), autoimmunity (Sharma and Bayry, 2015), and allergy (Li et al., 2016). The rarest of granulocytic cells, basophils, were first discovered in 1879 by Paul Erlich using conventional light microscopy (Chirumbolo, 2012), but their functions remained largely obscure until they were found to release histamine upon binding immunoglobulin E (IgE) via high-affinity IgE receptors (Ishizaka et al., 1972, 1973, 1979). Basophils account for approximately 0.5%–1% of all leukocytes in bone marrow and circulating in peripheral blood (Galli, 2000; Galli et al., 1984; Galli and Tsai, 2012; Gauvreau and Denburg, 2015; Gauvreau et al., 2009). Since their discovery, basophils have been established as effector cells in type 2 inflammatory responses, which include their production of IL-4, IL-5, IL-25, and TSLP, contributing to allergic manifestations and parasitic infections (Galli, 2000; Galli et al., 1984; Siracusa et al., 2013). In addition, basophil populations are increased in myeloproliferative neoplasms, such as chronic myeloid leukemia and primary myelofibrosis (Cervantes et al., 2009; Langabeer and Haslam, 2017).

Typically, basophils are immunophenotypically defined by expression of the IL-3 receptor (IL-3R or CD123), lack of HLA-DR, constitutive expression of FcεRI, and more recently reported by expression of the activation marker, CD203c (Salter et al., 2016a, 2016b). Human and mouse studies additionally exclude other cell subsets by specifying CD49b^+^, CD69^+^, Thy-1.2^+^, CD117^−^, CD19^−^, CD11c^+/−^, CD3^−^, B220^−^, NK1.1^−^ (i.e. *in silico* depletion of non-basophil cells). Upon stimulation, basophils increase their intracellular production of inflammatory mediators. Specifically, *in vitro* basophil studies commonly utilize IL-3 and anti-IgE to stimulate and activate basophils by signal transduction through CD123 and crosslinking of FcεRI (Galli, 2019), respectively, with subsequent upregulation of inflammatory mediator production (histamine, IL-4, IL-6, IL-13 (Salter et al., 2016b)) and activation marker expression (CD69, CD203c (Salter et al., 2016a)).

There has been a considerable amount of work done on characterizing mouse basophil heterogeneity in the past, and there has been a debate regarding whether mouse basophils were indeed similar to human basophils (Dvorak, 2000; Lee and McGarry, 2007). The basophil heterogeneity observed needs to be studied further, to understand phenotypic, morphological, and functional heterogeneity in basophils (Oetjen et al., 2016; Siracusa et al., 2012). Researchers have attempted to understand basophil heterogeneity through transcriptomic profiling, but there still has not been any direct links from this transcriptomic work to basophil phenotypic heterogeneity (Chhiba et al., 2017; Hamey et al., 2019; MacGlashan, 2015; Monaco et al., 2019). Furthermore, there have been few studies exploring the proteomic, phenotypic profiles of human basophils using high-dimensional methods.

To meet this need, cytometry by time-of-flight (CyTOF or mass cytometry) is able to simultaneously measure over 40 proteins at single-cell resolution, enabling the evaluation of complex marker combinations and characterizing better the heterogeneity of circulating leukocytes. Indeed, mass cytometry has been used to find better strategies for identifying novel cell types and discovering novel cell subpopulations (Bendall et al., 2011, 2012, 2014; Hartmann et al., 2018). Some studies have evaluated the validity of using CyTOF to evaluate functional responses of basophils to more traditional stimulations (anti-IgE or IL-3) (Mukai et al., 2017; Tordesillas et al., 2016); however, no studies have focused on searching for new subpopulations of human basophils and their responses.

Taken together, much remains to be learned about human basophil biology and function, and employing next-generation technologies such as mass cytometry is valuable for understanding unique proteomic features of this rare cell type. Accordingly, we sought to explore the heterogeneity of circulating basophils in healthy people using mass cytometry, by targeting an array of common cell-surface molecules known to be variably expressed across granulocytes (Table 1). This mass-cytometry-based approach revealed phenotypically and functionally distinct subpopulations of phenotypically basophilic granulocytes (PBG; i.e, CD45^+^HLA-DR^−^CD123^+^ granulocytes), based on individual combinations of surface marker expression, and their responses to anti-IgE or IL-3 stimulation. Notably, one of the anti-IgE or IL-3 unresponsive PBG subpopulations showed classic neutrophil morphology when isolated using fluorescence-activated cell sorting (FACS). Given the consistency of these observations across different individuals, we believe that our findings help to define a new baseline for understanding of human basophil biology, which may stimulate new investigations to explore unappreciated functions of these phenotypically basophilic granulocyte (PBG) subpopulations.

**Table 1:** Granulocytic Targets Used for Mass Cytometry, Clones of Antibodies, Functional Description, and Population Distributions

Target	Clone	Description/Function	Distribution	References
CD203c (E-NPP3)	NP4D6	Transmembrane ectoenzyme; clearance of extracellular nucleotides	B	(Hennersdorf et al., 2005), (Buhring et al., 2001)
CD13	WM15	Transmembrane zinc metallopeptidase; cytokine processing	B N	(Hennersdorf et al., 2005), (Terstappen et al., 1990a), (Stroncek et al., 1998)
CD193 (CCR3)	5 × 10^8^	G-protein coupled transmembrane receptor; chemokine receptor	B E	(Uguccioni et al., 1997), (Daugherty et al., 1996)
CD14	M5E2	Transmembrane glycoprotein; LPS receptor	B N E	(Iida et al., 1994), (Terstappen et al., 1990a)
CD244 (2B4)	C1.7	CD2 family transmembrane receptor; inhibition and activation of NK cells	B E	(Hennersdorf et al., 2005), (Munitz et al., 2005)
FcεRIa	CRA-1	Ig superfamily, transmembrane receptor; high affinity IgE receptor	B	(Valent, 1994)
CD123	6H6	Transmembrane glycoprotein; IL3 receptor	B	(Hennersdorf et al., 2005), (Agis et al., 1996)
CD44	IM7	Adhesion molecule; immune memory and activation	B N E	(Valent et al., 1990), (Mikecz et al., 1995)
MRP-14 (calgranulin B)	MRP 1H9	S100 family of protein; calcium-dependent activation	N	(Hessian et al., 1993)
CD15	W6D3	Poly-N-acetyllactosamine; adhesion molecule	N E	(Skubitz and Snook, 1987), (Terstappen et al., 1990b)
CD16	3G8	Ig superfamily, transmembrane receptor; low-affinity IgG receptor	B N	(Terstappen et al., 1990b), (Huizinga et al., 1988), (Meknache et al., 2009)
CD11b	M1/70	Transmembrane glycoprotein; adhesion molecule, chemotaxis, neutrophil activation	N	(Mann and Chung, 2006), (Terstappen et al., 1990b), (de Haas et al., 1994)
CD66b	80H3	Transmembrane glycoprotein; adhesion molecule	N	(de Haas et al., 1994), (Skubitz et al., 1995)
CD116 (GMCSF-r)	4H1	Transmembrane glycoprotein; cell proliferation, differentiation	B N E	(Valent, 1994), (DiPersio et al., 1988)
CD33	WM53	Transmembrane glycoprotein; adhesion molecule	B N E	(Terstappen et al., 1990a)
CD305 (LAIR-1)	NKTA255	Transmembrane protein; inhibition of cell cytotoxicity, cell activation, proliferation, and differentiation	B E	(Verbrugge et al., 2006), (Meyaard, 2008)
CD53	HI29	Transmembrane tetraspan family receptor; signal transduction, B cell activation	N E	(Matsumoto et al., 1999), (Mollinedo et al., 1998)
CD88 (C5aR)	S5/1	C5a receptor; chemotaxis, granule enzyme release, and superoxide anion production	N E	(Hugli and Muller-Eberhard, 1978), (Gerard et al., 1989)
CD183	CXCR3-173	G protein-coupled transmembrane receptor; CXC-chemokine receptor	E	(Jinquan et al., 2000)
CD191 (CCR1)	TG4/CCR1	G-protein coupled transmembrane receptor; cell migration	B E	(Florian et al., 2006; Ponath et al., 1996)
CD294 (CRTH2)	BM16	Seven-transmembrane protein coupled with G proteins, chemotaxis	B E	(Hirai et al., 2001)
CD52	HI186	Transmembrane glycoprotein	E	(Elsner et al., 1996)
CD49d (VLA-4)	9F10	Transmembrane glycoprotein; cell trafficking and inflammation	E	(Dobrina et al., 1991), (Sriramarao et al., 1994)
CD7	CD7-6B7	Ig superfamily transmembrane glycoprotein; adhesion molecule	NK T	(Rabinowich et al., 1994)

Our antibody panel is composed of lineage markers and proteins that have been reportedly expressed under homeostasis or generally associated with granulocyte activation. B = basophils, E = eosinophils, N = neutrophils, NK = NK cells, and T = T cells. See also Table S1.

## Results

### Mass Cytometry Reveals Four Distinct Subpopulations within “Classic” Human Basophils

**Figure 1:**
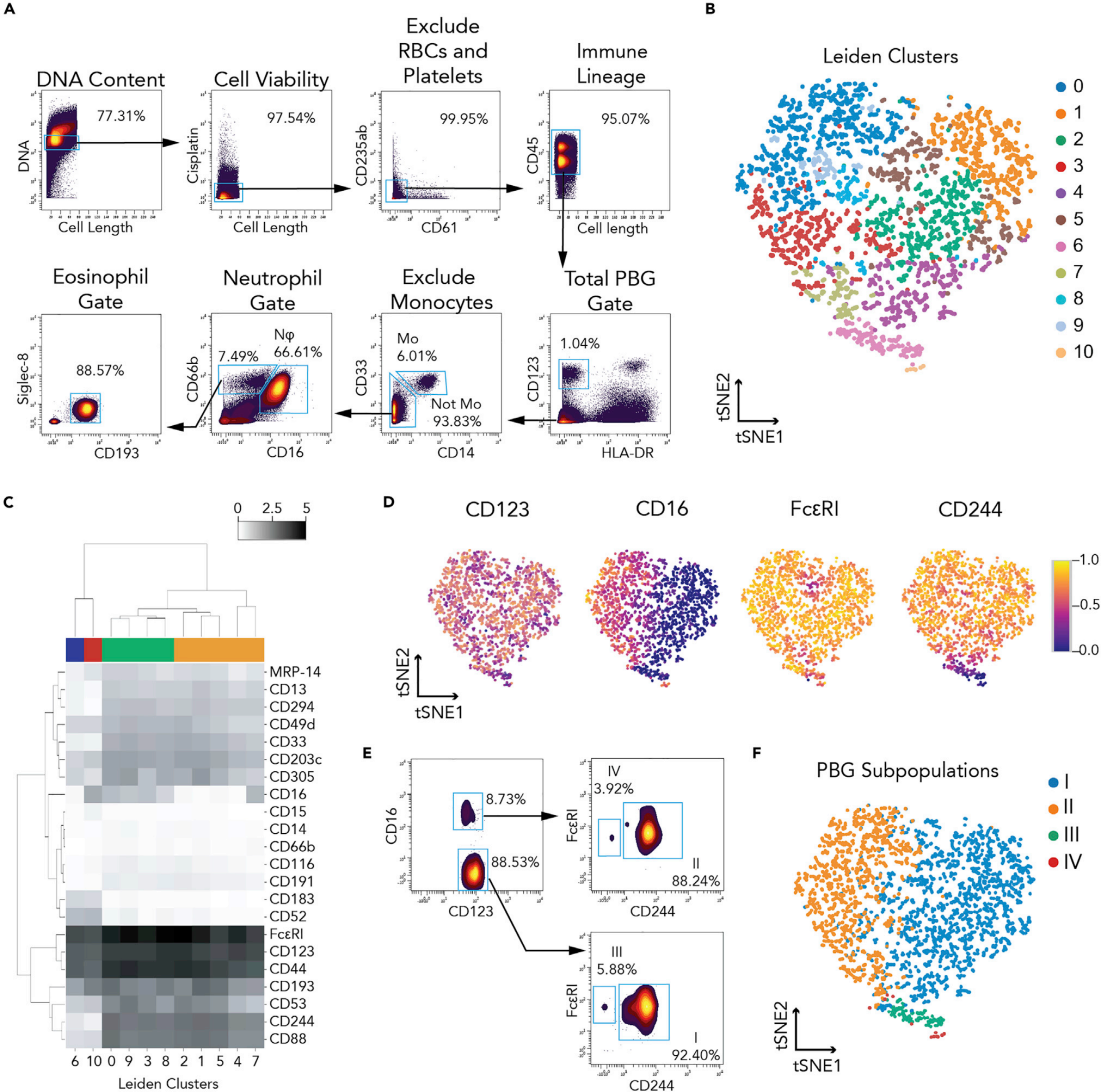
Mass Cytometry Analysis Reveals Four Phenotypically Basophilic Granulocyte Subpopulations Identified by Traditional CD45^+^HLA-DR^−^CD123^+^ Gating (A) Gating strategy plots for total phenotypically basophilic granulocytes (PBGs, CD45^+^HLA-DR^-^CD123^+^), eosinophils (CD45^+^HLA-DR^−^CD123^−^CD14^−^CD66b^+^CD193^+^Siglec8^+^), and neutrophils (CD45^+^HLA-DR^-^CD123^−^CD14^−^CD66b^+^CD16^+^) using classic lineage markers. (B) Implementation of Leiden clustering algorithm using the following markers: CD123, CD16, FcεRI, CD244, CD53, CD305, and CD193. (C) Four donors' mean median expression (arcsinh with cofactor 5 transformation) across clusters identified by the Leiden algorithm, hierarchically clustered by protein expression and by Leiden clusters. (D) Normalized single cell expression across of the following proteins: CD123, CD16, FcεRI, and CD244. (E) CD16, FcεRI, and CD244 used to manually draw gates in biaxial plots to distinguish the four PBG subpopulations. (F) The four-gated PBG subpopulations superimposed on the original tSNE plot coordinates. See also Figures S1 and S2.

To capture the heterogeneity of healthy human granulocytes, we assembled a multiplexed, mass cytometry antibody panel against proteins previously reported to be expressed on granulocytes under homeostasis or activation, with a particular focus on the basophil compartment (Tables 1 and S1). In this antibody panel, we included cell-surface markers that allowed us to identify the three granulocyte subsets (basophils, eosinophils, and neutrophils) using widely published, traditional gating schemes (Gustafson et al., 2015; Yu et al., 2016) (Figure 1A).

We collected whole peripheral blood from 19 healthy donors (Table S2) and handled samples consistently, under the same conditions as previously published (Mukai et al., 2017), and profiled protein expression in basophils, eosinophils, and neutrophils. Across these granulocyte subsets, we found that protein expression was heterogeneous. Basophils showed variability across upward of 20 protein markers (Figures S1 and 1), whereas eosinophils showed variability in expression of CD44, CD88, and CD16 (Figure S2A). Neutrophils also showed heterogeneity in MRP-14 expression (Figure S2C).

To identify phenotypically distinct cell subsets and their combinatorial expression patterns for heterogeneous markers in each granulocyte population, we employed tSNE (Amir et al., 2013; van der Maaten and Hinton, 2008). Briefly, tSNE separates cells by their quantitative expression of user-specified markers, reducing the many dimensions of these markers onto a two-dimensional plot. Cells with similar marker expression values are nearby one another on this plot, whereas cells with markedly different values are far away from each other, thus conserving the multidimensional data structure (Amir et al., 2013). We identified a subset of proteins (CD16, FcεRI, CD244, CD53, CD305, and CD193) that were able to capture the majority of PBG heterogeneity by tSNE analysis, which consistently demonstrated at least three distinct lobes across donors (Figure S1). In addition, we used the Leiden (Traag et al., 2019) clustering algorithm to identify subpopulations within CD45^+^HLA-DR^-^CD123^+^ cells based on the expression of CD16, FcεRI, CD244, CD53, CD305, and CD193. Eleven subpopulations were generated through the Leiden algorithm (Figure 1B), which we then metaclustered into four subpopulations based on their expression of 22 proteins (Figures S1B and 1C). However, we were not able to consistently reproduce subpopulations of classical blood eosinophils (Figure S2B) or neutrophils (Figure S2D).

PBGs showed heterogeneous expression across a variety of markers, with CD16, FcεRI, and CD244 capturing the majority of heterogeneity in these PBG subpopulations (Figure 1D). CD16, FcεRI, and CD244 were sufficient to manually draw gates in biaxial plots to distinguish the four PBG subpopulations (Figure 1E). These gates were then superimposed onto the tSNE map to determine the tSNE coordinates of the subpopulations (Figure 1F).

**Figure 2:**
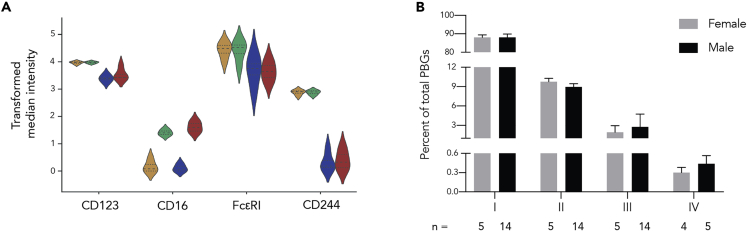
Characterization of Four Phenotypically Basophilic Granulocyte Subpopulations across Donors (A) Four donors' mean median expression (arcsinh with cofactor 5 transformation) for CD123, CD16, FcεRI, and CD244 shown across basophil subpopulations. (B) Plot indicating mean abundance and standard error of each cluster in the “classic” basophil gate across female and male blood donors. There were not any statistically significant differences between female and male basophil subpopulation abundances. See also Table S2.

With traditional cytometry gating by expression of CD16, FcεRI, and CD244, we proceeded to identify PBG subpopulations and their frequencies across all healthy donors. All of the PBG subpopulations showed high expression of CD123, a traditional basophil marker that is used consistently to classify cells as basophils in cytometry (Mukai et al., 2017; Salter et al., 2016a). Subpopulation I was CD16^low^FcεRI^high^CD244^high^, subpopulation II was CD16^high^FcεRI^high^CD244^high^, subpopulation III was CD16^low^FcεRI^low^CD244^low^, and subpopulation IV was CD16^high^FcεRI^low^CD244^low^ (Figure 2A). Subpopulation I comprises the bulk of all basophils, whereas subpopulation IV constitutes the fewest of these already rare cells. Furthermore, with the exception of subpopulation IV, there was little variation in the mean subpopulation abundance across donors (Figure 2B). Compared with basophil subpopulation I, subpopulation II was approximately 9-fold lower, subpopulation III 35-fold lower, and subpopulation IV 90-fold lower. However, the average proportions of each PBG subpopulation were similar between males and females. Interestingly, when separating donors by sex, we found that 4 out of 5 (80%) female donors had population IV, in contrast to only 5 out of 14 (36%) male donors.

### Rare CD16^high^FcεRI^low^CD244^low^ PGB (Subpopulation IV) Has Neutrophilic Morphology

**Figure 3:**
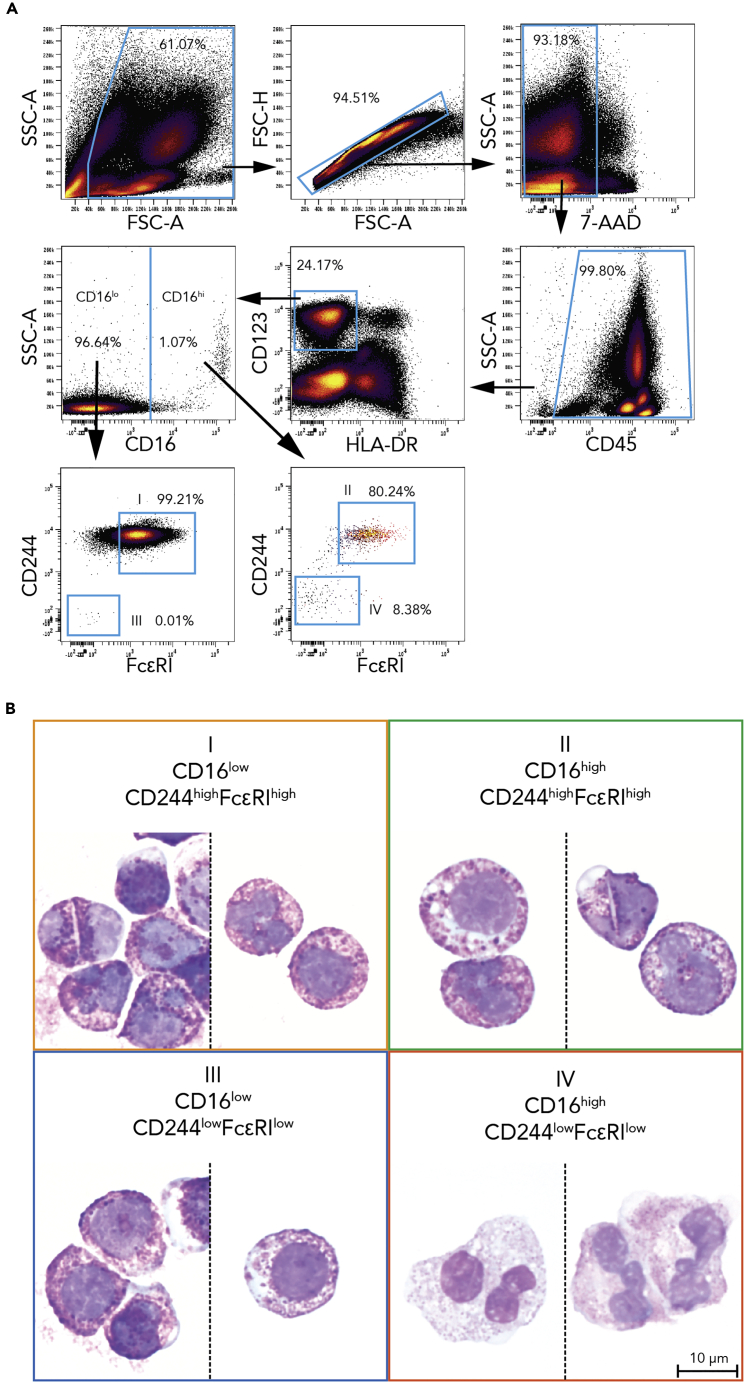
Three Out of Four Phenotypically Basophilic Granulocyte Subpopulations Are Morphologically Basophils (A) The CD45^+^HLA-DR^−^CD123^+^ gate traditionally assigned as basophils was analyzed for CD16 and split into high and low CD16 levels. These two CD16 sub-gates were then analyzed for CD244 and FcεRI levels. Subpopulations were identified as follows: subpopulation I as CD16^low^CD244^high^FcεRI^high^, subpopulation II as CD16^high^CD244^high^FcεRI^high^, subpopulation III as CD16^low^CD244^low^FcεRI^low^, and subpopulation IV as CD16^high^CD244^low^FcεRI^low^. (B) Cytocentrifugation, Wright-Giemsa staining, and light microscopy revealed that basophil subpopulations I, II, and III appeared morphologically to be basophils, but cluster IV showed typical neutrophil morphology. Colored frames correspond to subpopulation colors in Figure 2.

All PBG subpopulations expectedly expressed CD123 uniformly; however, PBG subpopulations III and IV showed unexpectedly lower expression of the traditional basophil marker FcεRI. Thus, we sought to confirm the identities of all of the PBG subpopulations by orthogonal means to better understand this uncovered basophil heterogeneity. We therefore used conventional fluorescent-activated cell sorting (FACS) to isolate each population based on their identified cell-surface phenotype into its own tube and cytocentrifuged the cells in each tube onto separate glass slides for light microscopic evaluation by a board-certified and trained hematopathologist (A.G.T.). Subpopulations I and II, which had high expression of FcεRI (Figure 2D), showed the coarse, basophilic granules that help to define basophils (Figure 3B, panels I and II correspond to each population). However, subpopulations III and IV, which had low FcεRI, showed distinctly different morphologies. Subpopulation III showed typical basophilic morphology (Figure 3B, panel III), but subpopulation IV showed typical features of neutrophils—fine, pink granules and more frequently tri-lobate nuclei (Figure 3B, panel IV).

### Contrasting Basophil Subpopulations to Traditional Granulocyte Fractions

**Figure 4:**
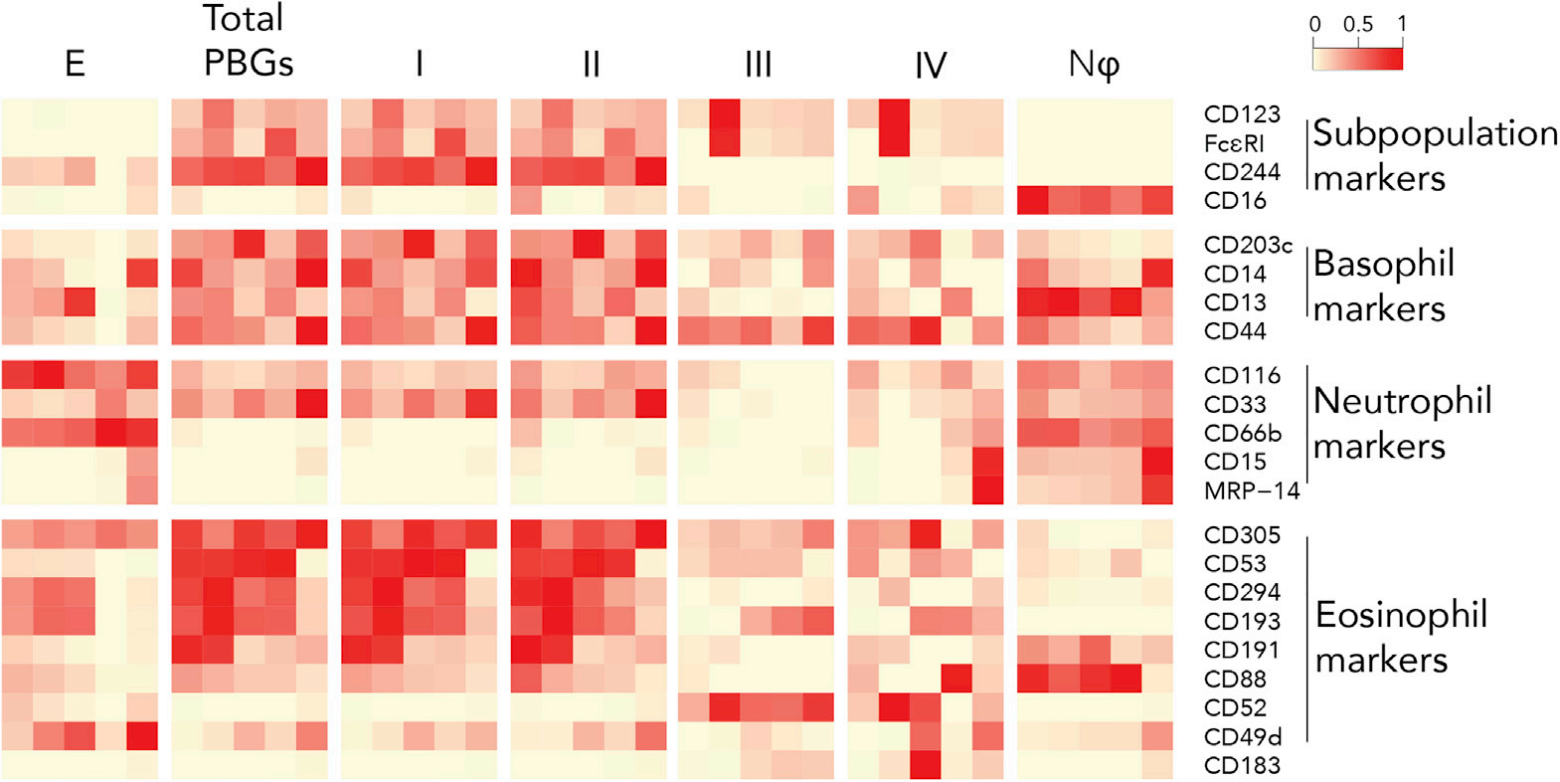
Phenotypic Profile of “Basophil” Subpopulations Compared with Traditional Granulocyte Populations A heatmap summary of median protein marker levels detected across eosinophils (E), total PBG (CD45^+^HLA-DR^−^CD123^+^) (B), PBG subpopulations (I, II, III, and IV), and classical neutrophils (Nϕ). Protein markers were organized by their use as clustering markers and by their known expression (Table 1) across traditional granulocyte subpopulations. Marker medians were transformed by calculating the difference of inverse hyperbolic sine (arcsinh) and normalized by individual markers. Columns indicate the five donors that were compared across granulocyte populations.

To contrast our four PBG subpopulations with the three traditionally defined populations of granulocytes—bulk basophils, eosinophils, and neutrophils—we compared their surface protein expression. Despite their distinct morphological features (Figure 3B), subpopulations III and IV showed remarkable similarities in granulocyte-associated protein expression. Two of the subpopulation-defining markers, FcεRI and CD244, showed lower expression in PBG subpopulations III and IV, making these PBGs more like neutrophils than “classic” basophils. However, FcεRI expression was higher than in most neutrophils. Because PBG subpopulation IV was morphologically neutrophilic (Figure 3B), we expected the expression of tested granulocytic markers to be similar to that of traditional neutrophils; however, we observed inconsistent, low expression of neutrophil markers in our panel (CD116, CD33, CD66b, CD15, and MRP-14), including CD16 (Figure 4). The other subpopulation-distinguishing marker, CD16, was most highly expressed in neutrophils compared with other granulocytes. Inadvertently, our normalization of protein levels that we used to generate the heatmap in Figure 4 obscured the differences in CD16 levels within the PBG subpopulations.

### Identifying Basophil Populations Responsive and Unresponsive to Anti-IgE or IL-3 Stimulation

**Figure 5:**
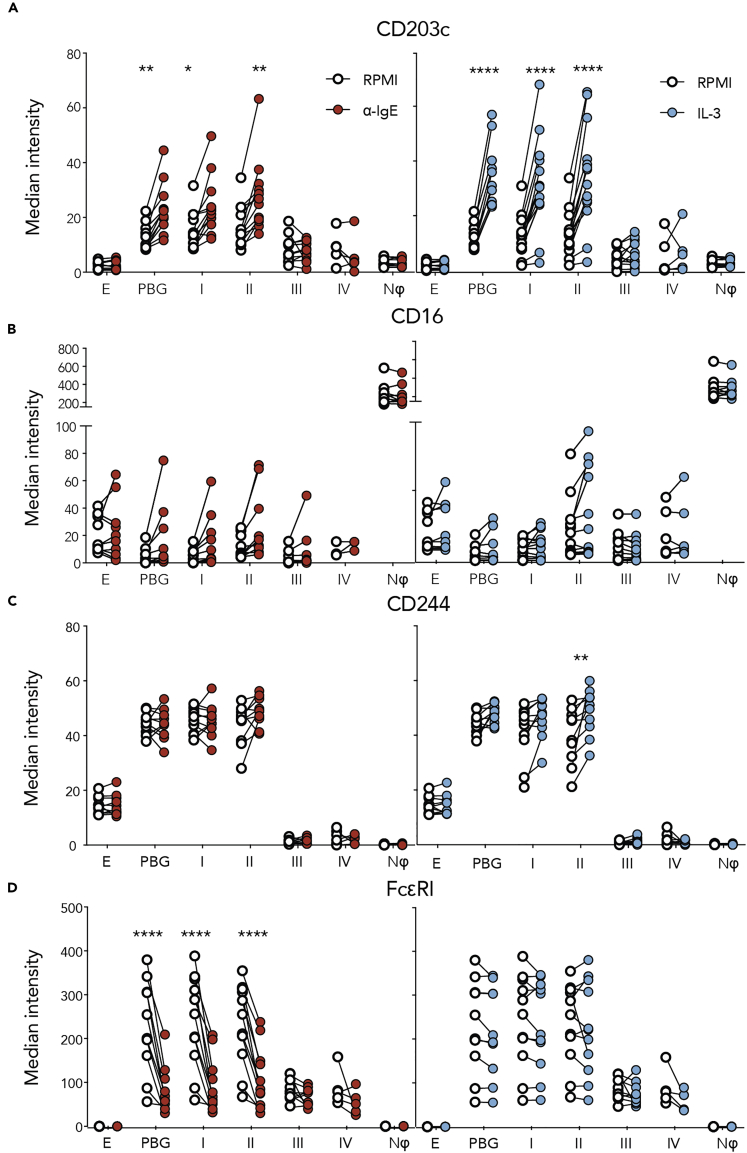
Functionality of Phenotypically Basophilic Granulocyte Subpopulations Compared with Traditional Granulocyte Subpopulations The CD203c basophil activation marker (A) was used to examine the potential for activation across PBG subpopulations (I, II, III, and IV) compared with total PBGs, neutrophils (Nϕ), and eosinophils (E). Stimulations were carried out in response to anti-IgE (red) or IL-3 (blue) stimulation compared with control RPMI media (open circles). Protein markers CD16 (B), CD244 (C), and FcεRI (D), used to delineate the four PBG subpopulations, were also analyzed to determine their modulation upon anti-IgE or IL-3 stimulus. Significant findings are denoted by ∗, ∗∗, and ∗∗∗∗ where p < 0.05, <0.01, and <0.0001 compared with values for RPMI controls. See also Figure S3.

To determine the functionality of the four basophil subpopulations, we determined the expression of CD203c, a classic basophil activation marker, across PBG subpopulations and traditional granulocytes after anti-IgE or IL-3 stimulation (Figure 5A). Like “classic” basophils, subpopulations I and II showed a significant two-fold upregulation in CD203c in response to anti-IgE or IL-3, indicating immune activation. However, PBG subpopulation III and subpopulation IV did not significantly upregulate CD203c, and generally had a three-fold lower expression compared with subpopulations I and II after anti-IgE or IL-3 stimulation. Thus, PBG subpopulation III and subpopulation IV, the neutrophil-like cells, appeared inactive to traditional basophil activators, behaving more like neutrophils.

Furthermore, we sought to determine whether the expression of basophil subpopulation markers CD16 (Figure 5B), CD244 (Figure 5C), and FcεRI (Figure 5D) was consistent across immune stimuli. Of the 19 donor samples, 2 increased CD16 expression in response to anti-IgE in subpopulations I, II, and III, but the increases were not significant. In PBG subpopulation II, CD16 expression also increased in response to IL-3; however, these changes were not significant and were present in only 4 donor samples (Figure 5B). Although CD244 did not change upon anti-IgE stimulation, subpopulation II PBGs significantly increased in CD244 expression in response to IL-3 (Figure 5C). Anti-IgE stimulation significantly decreased FcεRI detection (Figure 5D). However, this can be explained by steric hindrance of the anti-IgE antibody used for stimulation, which binds FcεRI and therefore blocks the antibody staining of FcεRI. In addition, this decrease in FcεRI can be explained by internalization of FcεRI after anti-IgE crosslinking (Molfetta et al., 2014). IL-3 stimulation did not change FcεRI levels (Figure 5D).

Because subpopulations III and IV displayed a unique phenotypic profile (Figure 4) and subpopulation IV showed neutrophilic morphological features (Figure 3B), we also evaluated how other granulocytic surface markers responded to anti-IgE or IL-3 stimulation across the PBG subpopulations. Although we observed inconsistent changes in the granulocyte-associated markers CD33, CD116, CD66b, CD15, and MRP-14 (Figures S3B–S3F), CD13 was significantly upregulated in “classic” basophils as well as subpopulations I and II (Figure S3A).

### PGB Subpopulation Distributions Are Similar between Peripheral Blood and Bone Marrow and Conserved in Chronic Myeloid Leukemia-Related Basophilia

**Figure 6:**
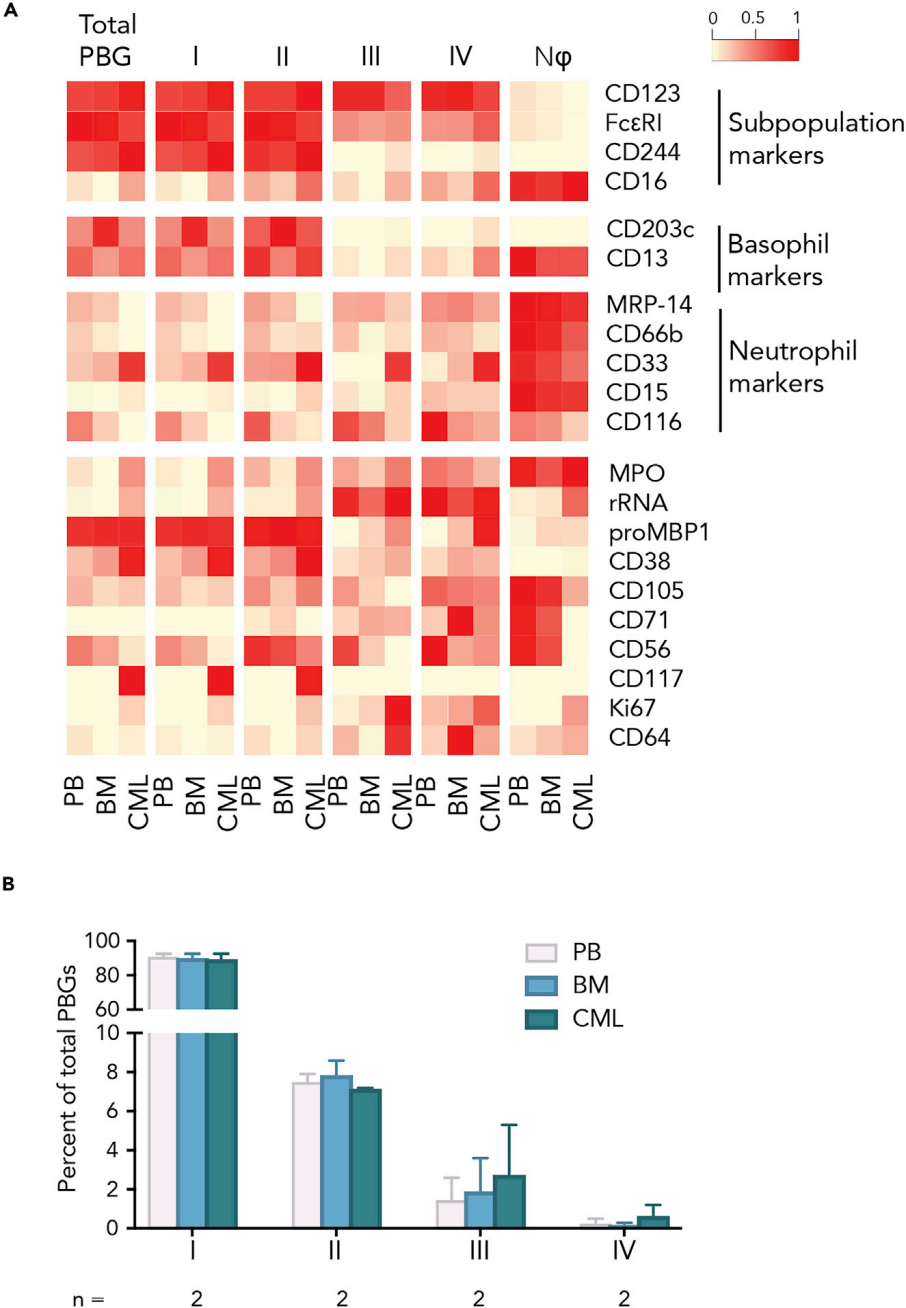
Profile of Phenotypically Basophilic Granulocyte Subpopulations in Healthy and CML Samples, across Compartments A heatmap summary of median marker levels detected in PBG subpopulations compared with bulk PBG basophils and neutrophils. Markers include granule specific proteins important in basophil functional profiles. Marker medians were first transformed by calculating the difference of inverse hyperbolic sine (arcsinh), followed by normalization by individual markers. (A) Total PBG basophils, the four identified subpopulations (I, II, III, and IV), and neutrophils (Nϕ) were profiled in paired samples of peripheral blood (PB) and bone marrow (BM) in healthy donors and compared with bone marrow from donors with CML. (B) Plot indicating abundance of each subpopulation in the total PBG gate across peripheral blood, bone marrow, and bone marrow of CML donors.

To determine the stability of basophil subpopulations across immune compartments, we compared paired samples of peripheral blood and bone marrow samples collected from healthy individuals. In addition, we used bone marrow from patients with chronic myeloid leukemia (CML) to determine whether the distribution or phenotype of PBG subpopulations is perturbed in a disease with characteristic basophilia (Figure 6). In addition to examining the persistence of the basophilic and neutrophilic surface markers across the basophil subpopulations, we also probed basophils to detect granule-specific proteins important in granulocytic functional profiles.

The phenotypic profiles of the four PBG subpopulations were consistently similar across peripheral blood, bone marrow, and CML bone marrow samples. In addition to the unique phenotypes of subpopulations III and IV previously described (Figure 4), we observed myeloperoxidase (MPO) expression, a lysosomal enzyme, specifically in subpopulation IV. MPO is typically produced by neutrophils, and our neutrophil-like cell population expressed this protein. Inversely, the level of the proform of human eosinophil granule major basic protein 1 (proMBP1), a published marker for basophil identification (Plager et al., 2006), was high in total PBGs and subpopulations I and II but low in subpopulations III and IV, like in neutrophils. In contrast to total PBGs, subpopulations I and II, and neutrophils, subpopulations III and IV expressed high levels of rRNA and Ki67, which were detected and consistently similar across peripheral blood, bone marrow, and CML samples (Figure 6A). Similar to that shown in Figure 2E, in peripheral blood as well as bone marrow and CML bone marrow samples, compared with PBG subpopulation I, subpopulation II was 9-fold lower, subpopulation III was 35-fold lower, and the neutrophil-like subpopulation IV was 90-fold lower (Figure 6B). Furthermore, the distribution of the four CD45^+^HLA-DR^-^CD123^+^ or PBG subpopulations remained consistently stable across the immune compartments and conditions tested.

## Discussion

The heterogeneity of most human granulocytes, including neutrophils and eosinophils, has been widely studied, elucidating their many functions within human health and disease (Cromheecke et al., 2014). However, studies focusing on basophils have often been limited due to their rarity (<1%) in human blood. Despite this limitation, the field of basophil biology has been rich in elucidating their function in inflammation, autoimmunity, and cancer. Cytometry has been a great tool in understanding these rare cell types; specifically, conventional flow cytometry has traditionally been used to immunophenotype basophils (Han et al., 2008; Mukai et al., 2017; Stacchini et al., 2011), and a few studies utilizing mass cytometry have also been done to interrogate the basophil compartment (Mukai et al., 2017; Tordesillas et al., 2016). Mukai et al. and Tordesillas et al. showed that basophils were able to be characterized and immunophenotyped using mass cytometry. Specifically, they confirmed basophil activity in their peanut-allergic patients by demonstrating upregulated CD63 and CD203c expression (Mukai et al., 2017; Tordesillas et al., 2016). Based on their success in measuring this rare cell population with mass cytometry, we sought to specifically profile basophils using mass cytometry, also referred to as cytometry by time-of-flight (CyTOF). This study was the first to use this mass cytometry technique to deeply study human basophils in multiple compartments (peripheral blood and bone marrow).

Using our methodologies and unique mass cytometry panel, the traditional CD45^+^HLA-DR^−^CD123^+^ phenotype for identifying putative basophils was revealed to contain four distinct phenotypically basophilic granulocyte subpopulations: (1) subpopulation I CD16^low^FcεRI^high^CD244^high^; (2) subpopulation II CD16^high^FcεRI^high^CD244^high^; (3) subpopulation III CD16^low^FcεRI^low^CD244^low^; and (4) subpopulation IV CD16^high^FcεRI^low^CD244^low^. Understanding that basophils are already only 0.5%–1% of all leukocytes, based on baseline levels alone, we found that subpopulation IV was approximately 90-fold smaller than subpopulation I.

Despite their low abundance, basophils play a vital role in disease pathobiology and elicit robust functional responses such as rapid release of granule proteins and histamine (Borriello et al., 2017; Youssef et al., 2007). Specifically, in allergic inflammation, basophils initiate the allergic cascade due to their rapid release of histamine and other mediators (Gibbs, 2008), and depletion and knock-out models of basophils have shown abrogation of disease pathogenesis (Matsuoka et al., 2013). Disruption of basophil function, as demonstrated by Watson et al. by targeting human basophils through their nicotinic acetylcholine receptor using ASM-024, inhibited IgE-mediated and allergen-induced activation of basophils demonstrated by a significant decrease in CD203c expression (Watson et al., 2014). Furthermore, upstream alarmin cytokines TSLP, IL-25, and IL-33 promote the onset of the type-2 inflammatory cascade, which increase basophil activation and migration potential (Salter et al., 2015, 2016a, 2016b). However, studies evaluating basophils have examined only the basophil populations as a whole or in bulk, with no attempt to further subcategorize this rare cell type.

PBG subpopulations I–III were consistently found in all donor samples, whereas the neutrophil-like subpopulation IV was only present in 9 of 19 donor blood samples analyzed (4 of 5 females and 5 of 14 males). Given that subpopulation IV is approximately 1% of conventional basophils, which themselves represent only 1% of leukocytes, we hypothesize that these 0.01% of leukocytes may either live transiently in the circulation or not be present at all in some individuals. Distinguishing between these possibilities will require more extensive studies. Furthermore, cell sorting and microscopy demonstrated that subpopulations I–III appeared morphologically to be typical basophils, whereas subpopulation IV appeared morphologically to be typical neutrophils. Interestingly, Smith et al. reported that neutrophils express transcripts of IL-3R (CD123), a classic surface marker used to gate basophils (Smith et al., 1995). One may speculate that subpopulation IV cells are neutrophils with some basophilic-type properties.

Based upon PBG surface-expression profiles, subpopulations I and II were most comparable to classic basophils and exhibit the most intra-heterogeneity (Figure S1B, and Figure 1C), whereas subpopulations III and IV were most comparable to classic neutrophils. Specifically, FcεRI and CD244, two of the markers distinguishing the basophil subpopulations, were lower in expression in basophil subpopulations III and IV, which were similar to the expression levels of classic neutrophils. Thus, basophil subpopulation III, despite its close morphological resemblance to classic basophils, had an expression profile similar to neutrophils. In contrast, the expression profiles of eosinophils did not resemble those of our PBGs, and microscopy did not show the coarse eosinophilic granules characteristic of eosinophils. From a developmental perspective, neutrophils (Gorgens et al., 2013; Paul et al., 2000) are thought to be derived from granulocyte-myeloid progenitors (GMP), whereas eosinophils and basophils (Gauvreau and Denburg, 2005, 2015; Gauvreau et al., 2009; Reece et al., 2014) are believed to be derived from a common eosinophil-basophil progenitor. Given that one of the PBG subpopulation-identifying markers, CD244, is functionally expressed on human eosinophils, it is interesting that PBG subpopulations would have more apparent overlap with neutrophils than with eosinophils (Munitz et al., 2005). Furthermore, in the perspective article by Lee and McGarry, they highlight important work done where FcεRI^+^ mouse basophils had unconventional morphologies that was out of the normal definition of classic basophils, and thus these aberrances in basophil morphology are not only present in our human work, but also in previous mouse work (Dvorak, 2000; Gessner et al., 2005; Lee and McGarry, 2007; Min et al., 2004; Mukai et al., 2005; Voehringer et al., 2004).

By interrogating PBG functional responses through whole blood treatment with classic basophil stimulation agents, anti-IgE or IL-3, we found that basophil subpopulations I and II behaved similarly to classic basophils, increasing expression of CD203c after both anti-IgE or IL-3 stimulation, as has been reported previously (Mukai et al., 2017; Salter et al., 2015, 2016a, 2016b; Tordesillas et al., 2016). However, subpopulations III and IV were non-responders and had similar expression levels as classic neutrophils, suggesting roles other than classical IgE-mediated responses. In addition, although unchanged post-stimulation, the expression levels of mature neutrophil markers CD66b, CD15, and MRP-14 were consistently higher compared with classic eosinophils, basophils, and other PBG subpopulations. Interestingly, MacGlashan has evaluated the transcriptome profile of human basophils after stimulation with IL-3 and anti-IgE and found that stimulation with IL-3 influenced the basophil transcriptome profile the most due to IL-3's prominent role in basophil maturation. However, the transcriptomic profiles were not linked to specific basophil phenotypes, and it was noted by MacGlashan that the isolation process of basophils, although having great yield, purity, and viability, induced transcriptional variability (MacGlashan, 2015). Thus, proteomic profiling of basophils to understand heterogeneity in basophil phenotype, morphology, and function might be the more robust method, as it was shown by Mukai et al. that basophil isolation and sample preparation did not have effects on downstream basophil activation profiles on a protein level (Mukai et al., 2017).

Alternatively, we acknowledge that our data do not further evaluate temporal changes in PBG subpopulations identified, which means PBG subpopulations I–IV could potentially be part of the same cellular continuum. Specifically, the higher expression of rRNA and Ki67 alludes to III and IV potentially being in a different phase of cell cycle or maturation state (Lee and McGarry, 2007) because population III and IV also have lower levels of proMBP1, a marker that can specifically characterize basophils, compared with population I and II. These differences in rRNA, Ki67, and proMBP1 levels helps support the differential activation profiles we noted between subpopulations I and II versus III and IV, where III and IV were less responsive to IL-3 and anti-IgE stimulation, and had lower levels of proMBP1 and higher levels of Ki67 and rRNA.

After interrogating PBG subpopulations in peripheral blood, we wondered if there were differences between blood and bone marrow samples from the same donor. We also wondered whether basophilia associated with CML would affect PBG subpopulations (Masuda et al., 2015; Stacchini et al., 2011; Valent et al., 2018). However, we found no differences in surface marker expression or distribution of PBG subpopulations between peripheral blood, bone marrow, or bone marrow samples from a limited set of CML patients. However, we do acknowledge our limited number (n = 2) of CML samples that we evaluated in this study. The main purpose of evaluating the PBG subpopulations in CML samples was to determine whether the PBG subpopulations were detectable and in similar proportions in basophil-associated diseases, exemplified by CML, compared with healthy individuals.

Given this consistency, future studies evaluating subpopulation IV might benefit from using CML as a model given that its basophils are more abundant for study than normal. At the same time, it is suggested that basophils may have heterogeneous morphologies, as demonstrated in samples from CML patients reported on previously (Sreedharanunni et al., 2016; Stacchini et al., 2011). Interestingly, in a rare case of CML, Stracchini et al. found basophils in peripheral blood exceeded 70% but were not identifiable because of atypical morphology, yet the basophils were evident by flow cytometry with a CD123 expression, the classic basophil identifying marker (Stacchini et al., 2011). However, the atypical morphology of basophils in this case could be explained by agitation of basophils during handling or prolonged refrigeration of basophils. Still, based on our study, the phenotypic expression profiles and proportions of PBG subpopulations were stable across compartments and between samples from healthy people and a limited number of CML patients, but more work is needed to understand their roles in propagating disease.

This study represents a deep phenotypic dive into the proteomic heterogeneity of PBGs with single cell mass cytometry. Our findings provide a new perspective regarding human granulocyte diversity, particularly shedding light on basophil heterogeneity. We revealed four distinct subpopulations of CD45^+^HLA-DR^-^CD123^+^ basophil-like granulocytes (PBG) identified through variability in CD16, CD244, and FcεRI expression: (I) CD16^low^FcεRI^high^CD244^high^, (II) CD16^high^FcεRI^high^CD244^high^, (III) CD16^low^FcεRI^low^CD244^low^, and (IV) CD16^high^FcεRI^low^CD244^low^. Although cell sorting and light microscopy showed that subpopulations I–III appeared morphologically to be classic basophils and subpopulation IV to be morphologically neutrophils, subpopulation III and IV shared similar expression profiles and functional activity to that of classic neutrophils. Our discovery of these unique neutrophil-like basophils and/or basophil-like neutrophils may provide new considerations for examining basophil and neutrophil behavior and function at the cellular level. These subpopulations could serve roles beyond traditional IgE-mediated immunity, which need to be interrogated further with additional functional characterization to shed light on their roles in health and disease.

### Limitations of the Study

There is still much to unravel about these phenotypically basophilic granulocytes, particularly subpopulation III and neutrophilic-like subpopulation IV. We have interrogated the proteomic, morphological, and activation profiles of these PBG subpopulations; however, their transcriptomic and metabolic profiles remain to be elucidated and is a strong future step to better understand the potential roles these PBGs play in health and disease. We also looked into sex differences in our PBG subpopulations, and although there were no proportional differences based on sex between subpopulations I and IV, in our study we found 80% of females and 36% of males had circulating PBG subpopulation IV, which could be of interest in basophilic diseases with sex-skewed prevalence. Furthermore, the developmental trajectory of PBGs from human bone marrow is yet another avenue to interrogate experimentally. Lastly, we successfully revealed our distinct subsets of basophils in bone marrow of CML patients; we only evaluated a limited number of these samples and thus made no conclusions on the roles these basophils subsets have in the pathogenesis of CML. Thus, future work can further evaluate PBGs in different basophilic-centric disease states to further delineate differential roles in PBGs.

### Resource Availability

#### Lead Contact

Further information and request for resources and reagents should be directed to and will be fulfilled by the Lead Contact, Dr. Sean Bendall (bendall@stanford.edu).

#### Materials Availability

The study did not generate new unique reagents.

#### Data and Code Availability

All relevant data and code are available from authors upon request.

## Methods

All methods can be found in the accompanying Transparent Methods supplemental file.

## Supplemental Information

Document S1. Transparent Methods, Figures S1–S3, and Tables S1 and S2
