# Supplementary material for: Mass Cytometry Phenotyping of Human Granulocytes Reveals Novel Basophil Functional Heterogeneity
Source: iScience. 2020 Oct 22;23(11):101724. doi: 10.1016/j.isci.2020.101724 (PMC7653073; doi:10.1016/j.isci.2020.101724)
Supplement: Document S1. Transparent Methods, Figures S1–S3, and Tables S1 and S2 [file mmc1.pdf]

iScience, Volume *23*

## Supplemental Information

# Mass Cytometry Phenotyping of Human Granulocytes Reveals Novel Basophil Functional Heterogeneity

**Nora Vivanco Gonzalez, John-Paul Oliveria, Dmitry Tebaykin, Geoffrey T. Ivison, Kaori Mukai, Mindy M. Tsai, Luciene Borges, Kari C. Nadeau, Stephen J. Galli, Albert G. Tsai, and Sean C. Bendall**

**SUPPLEMENTAL TABLES and FIGURES**

**Table S1. A summary of the antibodies used for our mass cytometry analysis**. Related to Table 1. The table shows the antigen (antibody target), antibody clone, the element and isotope the antibody was conjugated to, the final concentration of metal-conjugated antibody used for protein detection, and the vendor from whom we purchased the purified monoclonal antibodies.

| Antigen | Clone | Element | Mass | Desired final conc. (µg/mL) | Vendor |
|---|---|---|---|---|---|
| **CD3 (QD655)** | S4.1 | Cd | 112/114 | 3 | Invitrogen |
| **CD235ab** | HIR2 | In | 113 | 1 | BioLegend |
| **CD45** | HI30 | In | 115 | 1 | BioLegend |
| **CD61** | VI-PL2 | La | 139 | 0.5 | BioLegend |
| **CD7** | CD7-6B7 | Pr | 141 | 2 | BioLegend |
| **CD294** | BM16 | Nd | 143 | 2 | BioLegend |
| **CD191** | TG4/CCR1 | Nd | 144 | 4 | MBL International |
| **Siglec-8** | 7C9 | Nd | 145 | 4 | BioLegend |
| **CD164** | 67D2 | Nd | 146 | 4 | BioLegend |
| **CD20** | 2H7 | Sm | 147 | 2 | Fluidigm Sciences |
| **CD16** | 3G8 | Nd | 148 | 2 | Fluidigm Sciences |
| **CD13** | WM15 | Nd | 150 | 1 | BioLegend |
| **CD123** | 6H6 | Eu | 151 | 2 | BD |
| **CD66b** | 80H3 | Sm | 152 | 2 | Fluidigm Sciences |
| **FcεRI** | CRA-1 | Eu | 153 | 1 | BioLegend |
| **CD11b** | ICRF44 | Gd | 155 | 2 | BD |
| **CD183** | G025H7 | Gd | 156 | 2 | Fluidigm Sciences |
| **CD23** | EBVCS-5 | Gd | 157 | 8 | Sigma Aldrich |
| **CD33** | WM53 | Gd | 158 | 1 | Fluidigm Sciences |
| **CD116** | 4H1 | Tb | 159 | 2 | BioLegend |
| **CD14** | M5E2 | Gd | 160 | 2 | Fluidigm Sciences |
| **CD49d** | 9F10 | Dy | 162 | 1 | BioLegend |
| **MRP-14** | MRP 1H9 | Dy | 163 | 2 | Santa Cruz |
| **CD15** | W6D3 | Dy | 164 | 2 | Fluidigm Sciences |
| **CD52** | HI186 | Er | 166 | 1 | BioLegend |
| **CD53** | HI29 | Er | 167 | 3 | BD |
| **CD305** | NKTA255 | Er | 168 | 1 | Santa Cruz |

| | | | | | |
|---|---|---|---|---|---|
| **IgE** | MHE-18 | Tm | 169 | 1 | BioLegend |
| **CD44** | IM7 | Yb | 171 | 2 | Fluidigm Sciences |
| **CD203c** | NP4D6 | Yb | 172 | 0.5 | BioLegend |
| **CD244** | C1.7 | Yb | 173 | 1 | BioLegend |
| **HLA-DR** | L243 | Yb | 174 | 2 | Fluidigm Sciences |
| **CD193** | 5E8 | Lu | 175 | 4 | Fluidigm Sciences |
| **CD88** | S5/1 | Yb | 176 | 4 | BioLegend |
| **CD71** | CY164 | In | 115 | 1 | BioLegend |
| **CD105** | 43A3 | Nd | 146 | 8 | BioLegend |
| **proMBP1** | J175-7D4 | Sm | 149 | 0.25 | BioLegend |
| **CD56** | NCAM16.2 | Nd | 150 | 1 | BD |
| **MPO** | 1B10 | Dy | 161 | 0.05 | BD |
| **rRNA** | Y10b | Ho | 165 | 1.5 | Novus Biologicals |
| **CD38** | HIT2 | Er | 167 | 0.25 | BioLegend |
| **CD117** | 104D2 | Yb | 171 | 2 | BioLegend |
| **Ki67** | B56 | Lu | 175 | 2 | BD |
| **CD64** | 10.1 | Yb | 176 | 2 | BioLegend |

**Table S2. A summary of blood and bone marrow donors**. Related to Figure 2B. The table shows the sample type, sex and age of donors included in our study.

| Sample Type | Sex | Age |
|---|---|---|
| Peripheral blood | Male | 52 |
| Peripheral blood | Male | 61 |
| Peripheral blood | Male | 49 |
| Peripheral blood | Female | 51 |
| Peripheral blood | Female | 50 |
| Peripheral blood | Female | 62 |
| Peripheral blood | Male | 57 |
| Peripheral blood | Female | 57 |
| Peripheral blood | Male | 47 |
| Peripheral blood | Male | 43 |
| Peripheral blood | Male | 64 |
| Peripheral blood | Male | 19 |
| Peripheral blood | Female | 29 |
| Peripheral blood | Male | 69 |
| Peripheral blood | Male | 56 |
| Peripheral blood | Male | 60 |
| Peripheral blood | Male | 25 |
| Peripheral blood | Male | 54 |
| Peripheral blood and bone marrow | Male | 19 |
| Bone marrow | Male | 39 |
| CML bone marrow | Female | 53 |
| CML bone marrow | Male | 31 |

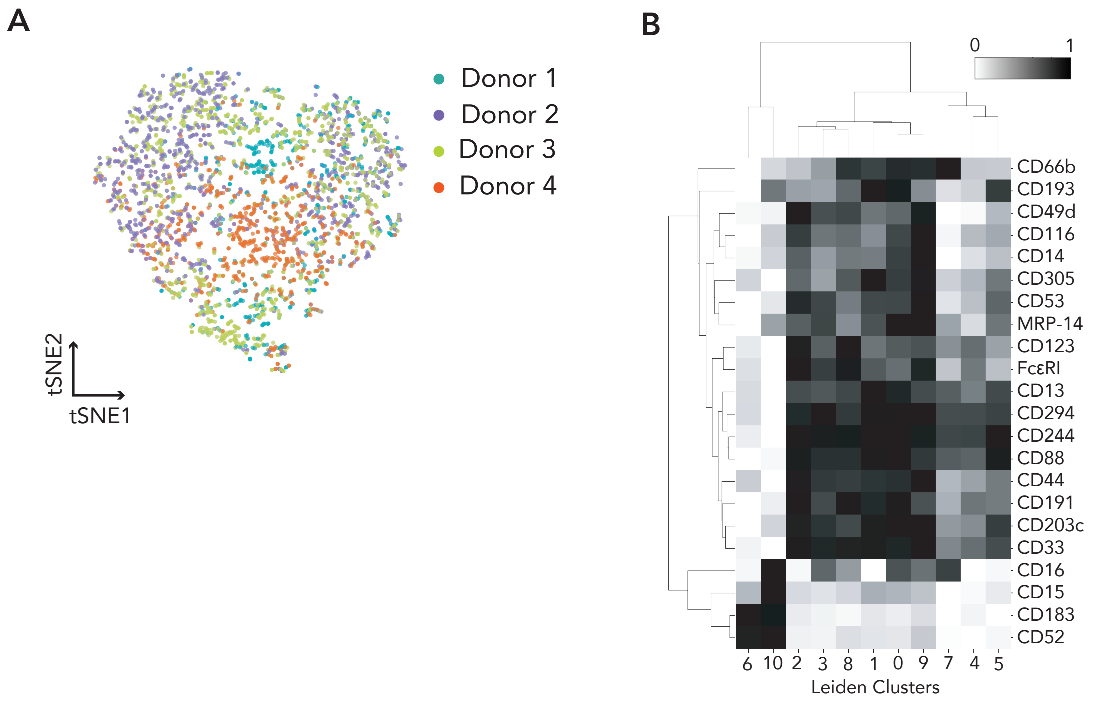


**Figure S1. Dimensionality reduction by tSNE and normalized protein expression on PBG Leiden clusters from four donors.** Related to Figure 1. (A) Donor basophil distribution overlaid on tSNE map from Figure 1B. (B) Normalized protein expression of four donors across PBG Leiden clusters was hierarchically clustered by protein expression and by Leiden clusters.

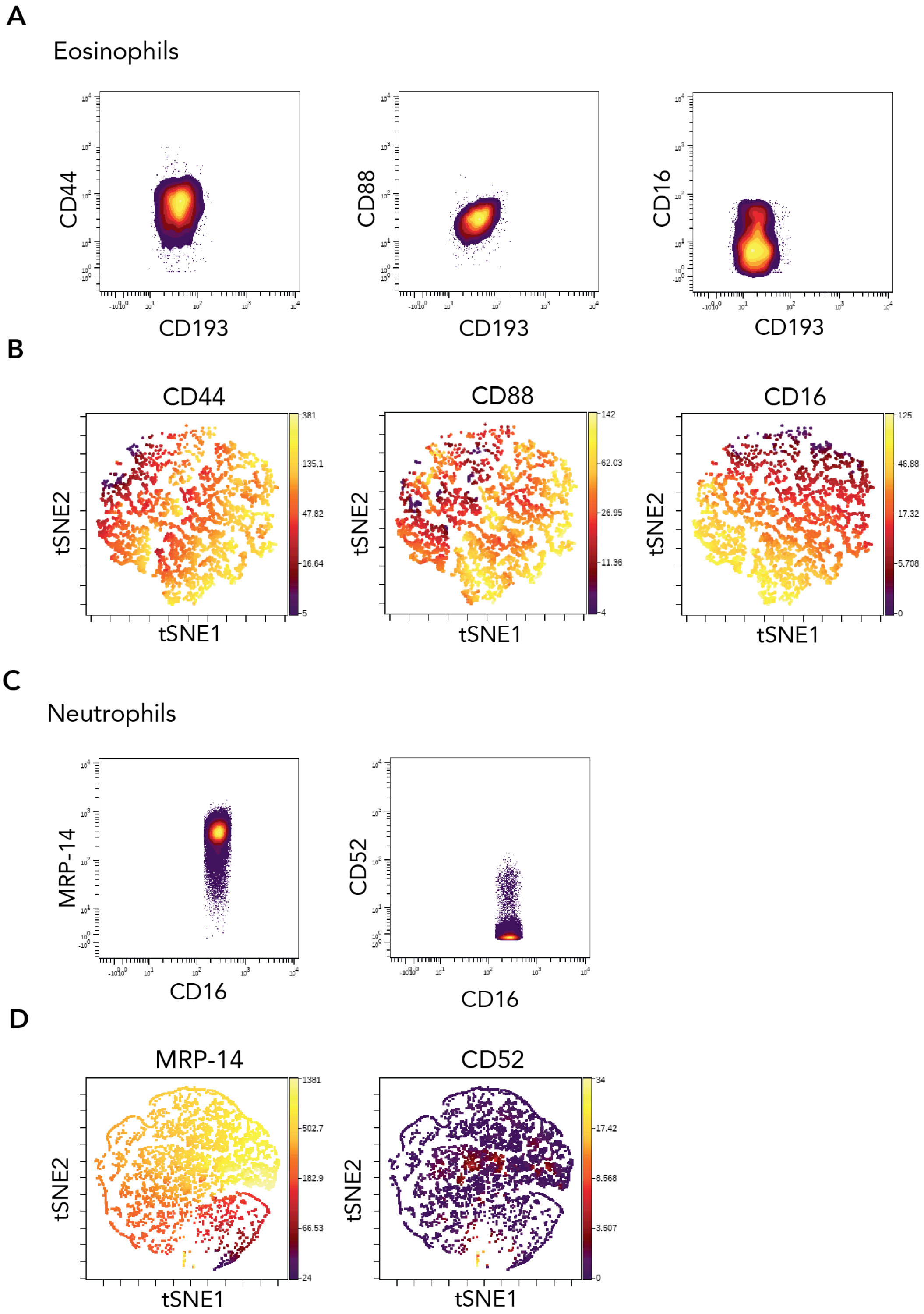


**Figure S2. Heterogeneity in human eosinophils and neutrophils.** Related to Figure 1. (A) CD44, CD88, and CD16 (on a representative biaxial plot) showed a range in expression on peripheral blood eosinophils. (B) These markers were selected for clustering analysis by tSNE, but we did not observe consistent eosinophil "islands" or subclusters across donors. (C) MRP-14 and CD52 (on a representative biaxial plot) showed heterogeneous expression on neutrophils. (D) We selected these markers for clustering analysis by tSNE, but we did not observe consistent neutrophil "islands" or subclusters across donors.

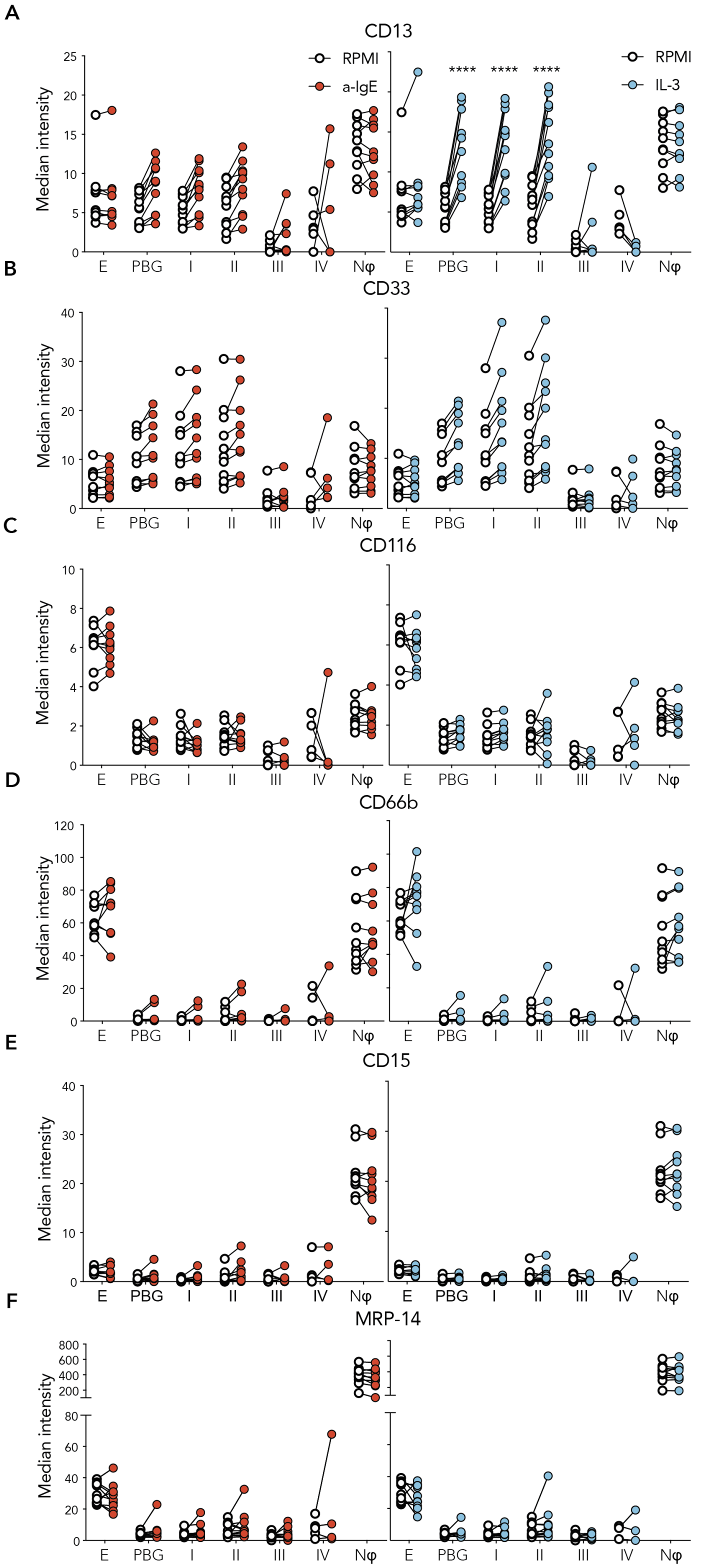


**Figure S3. Granulocyte-associated markers affected by anti-IgE or IL-3**. Related to Figure 5. PBG subpopulations (I, II, III, and IV), total PBGs, neutrophils (Nφ), and eosinophils (E) were stimulated with anti-IgE (red) or IL-3 (blue) and compared to cells exposed to RPMI media (open circles). The expression of (A) CD13, (B) CD33, (C) CD116, (D) CD66b, (E) CD15 and (F) MRP-14 were evaluated pre- and post-stimulation. Significant findings are denoted by **** where $p<0.0001$ compared to values for RPMI controls.

## TRANSPARENT METHODS

### *Specimen Collection*

Peripheral blood samples from anonymous healthy donors (with unknown allergy status) were obtained from the Stanford Blood Center (Palo Alto, CA, USA) via venipuncture in heparin- or EDTA-coated tubes and stored at 4°C for no more than 24 hours.

Healthy bone marrow and peripheral blood samples paired from the same donor were ordered from AllCells (Alameda, CA, USA) and delivered and processed the same day.

Samples from patients diagnosed with chronic myeloid leukemia (CML) were collected for diagnosis in EDTA or heparin tubes and stored at 4°C. Research aliquots were obtained <3 days after collection as post-diagnostic excess material under IRB-30899 and IRB-40765.

### *Antibodies*

A summary of all mass cytometry antibodies, reporter isotopes and concentrations used for analysis is displayed in Table S1. Except for CD3-QDot 655 (Invitrogen), primary conjugates of mass cytometry antibodies were prepared 200 µg at a time using the MaxPAR antibody conjugation kit (Fluidigm Sciences) according to the manufacturer's recommended protocol. After metal-labeling, antibodies were diluted in PBS-based Antibody Stabilization Solution (Boca Scientific) supplemented with 0.02% sodium azide to 0.2 mg/mL and stored long-term at 4°C. Each antibody clone and lot was titrated to optimal staining concentrations using human peripheral blood mononuclear cell samples.

### *Stimulation of Basophils*

One milliliter aliquots of whole blood were distributed into round-bottom tubes with loose lids and warmed in a 37°C water bath for 30 seconds. Freshly prepared, 1 mL aliquots of pre-warmed RPMI 1640 (Gibco) were supplemented with polyclonal rabbit anti-IgE (Bethyl Laboratories) or IL-3 (PeproTech). The pre-warmed 1 mL of whole blood was then mixed with either 1 mL of RPMI 1640, or RPMI 1640 supplemented with anti-IgE (final concentration, 1 µg/mL) or IL-3 (final concentration, 2 ng/mL) and incubated for 30 minutes at 37°C in a 5% $CO_2$ incubator. After the incubation period, red blood cells were removed by hypotonic lysis with 20 mL of cold RBC lysis buffer (BioLegend) and incubation for 15 minutes on ice. The lysis reaction was stopped with PBS supplemented with EDTA (Invitrogen Life Technologies; final concentration, 2 mM) followed by centrifugation at 250g for 5 minutes at 4°C (all centrifuge runs on live cells were done with these conditions).

### *Mass Cytometry Basophil Staining*

Cells were resuspended in cell staining medium (CSM; low barium PBS with 0.5% BSA, 0.02% sodium azide) and blocked with Human TruStain FcX (BioLegend) following the manufacturer's instructions. The corresponding surface antibody cocktail (Table S1) was then added yielding a 100 µL reaction volume and stained at room temperature for 30 minutes. Following surface staining, 1 mL of low barium PBS supplemented with Carboplatin (Sigma-Aldrich; final concentration, 0.5 µM) was added to the cell suspension as a viability stain and incubated at room temperature for 5 minutes. To stop the reaction, tubes were washed twice with cell staining medium. Following the wash steps, cells were fixed for 10 minutes at room temperature with formaldehyde (PFA; Electron Microscopy Sciences) at a final concentration of 1.6% (diluted in cell staining medium). Cells were then centrifuged at 500 g for 5 minutes at 4°C and washed once with cell staining medium prior to permeabilization with 4°C methanol for at 10 min at 4°C. Following cell permeabilization, cells were then washed twice in cell staining media and then stained with an intracellular antibody cocktail (see Table S1) in 100 µL at room temperature for 30 minutes. Cells were then washed with cell staining medium, stained overnight at 4°C with 1 mL of 1:4000 191/193 iridium DNA intercalator (Fluidigm Sciences), and diluted in low barium PBS with PFA at a final concentration of 1.6%. Prior to acquiring cells on the CyTOF2 mass cytometer (Fluidigm Sciences), cells were washed once with 4°C cell staining medium and twice with

double-deionized water ($ddH_2O$). Washed samples were then resuspended in $ddH_2O$ containing 1:10 dilution of EQ Four Element Calibration Beads (Fluidigm Sciences). Cells were acquired at an event rate of less than 500 cells per second.

***Mass cytometry data processing***

After cell acquisition, FCS files for each sample were processed through a MATLAB-based bead-normalization software (Finck et al., 2013) before being uploaded to Cytobank (Kotecha et al., 2010) for gating . All parameters except for time and cell length were displayed with an arcsinh transformation. As seen in Figure 1, events positive for intercalator-Ir were selected as having high DNA content. Cisplatin was then used to discriminate live from dead cells. Staining with CD235ab allowed exclusion of red blood cells from proceeding gates. Cells that exhibited high and medium levels of CD45 were then analyzed. Gating strategy plots for eosinophils ($CD45^+HLA\text{-}DR^-CD123^-CD14^-CD66b^+Siglec\text{-}8^+$), neutrophils ($CD45^+HLA^-DR^-CD123^-CD14^-CD66b^+CD16^+$) and basophils ($CD45^+HLA\text{-}DR^-CD123^+$) using classic lineage markers. We back-gated to make sure that none of the cells belonged in multiple gates.

Dimensionality reduction analyses were first conducted with viSNE in Cytobank, which uses the Barnes-Hut implementation of the tSNE algorithm. In order to compare multiple donors on the same dimensionality reduced plot, we downloaded a subset of the gated PGB data that contained a consistent antibody panel, and further processed with R (http://www.r-project.org) to quantile normalize protein expression by donor to correct for technical variation between CyTOF runs. Furthermore, we transformed the data with an inverse hyperbolic sine (arcsinh) transformation with a cofactor of 5 before employing Scanpy's (Wolf et al., 2018) Python-based (http://www.python.org) implementation.

We used Scanpy to carry out dimensionality reduction via tSNE and clustering with the Leiden algorithm. Up to 1,000 PBG from each of four donors were density sampled and used to generate a tSNE plot based on the expression of the following proteins: CD16, FcεRIα, CD244, CD53, CD305, and CD193. The Leiden algorithm was then used to cluster PGB based on the same proteins previously mentioned (Figure 1B).

***FACS Sorting and Cellular Morphometrics***

CD123+ cells were enriched from 10 mL peripheral blood tubes and leukocyte reduction chambers of healthy blood or platelet donors (Stanford Blood Center, Palo Alto, California) after red blood cell lysis and washing with cell staining medium (CSM, PBS, 0.05% BSA, 0.02% $NaN_3$) supplemented with 20 U/mL heparin and 25 U/mL benzonase (Sigma-Aldrich E8263), followed either by using αCD123 magnetic beads (Miltenyi Biotec, 130-094-432) on magnetic separation columns (Miltenyi Biotec, 130-042-201) or αCD123-biotin (Miltenyi Biotec 130-098-565) on magnetic streptavidin particles (BD 557812) per manufacturer's instructions. Enriched cells were then stained in a 100 µL reaction with 5 µL CD45 FITC (BioLegend 304006), 5 µL CD123 eFluor 450 (eBioscience 48-1239-41), 5 µL CD244 APC (BioLegend 329511), 1 µL CD16 PE/Cy7 (BioLegend 302015), 1 µL HLA-DR V500 (BD 561225), and 1.5 µL FcεRIα PE (BioLegend 334609), along with 5 µL Fc blocker (BioLegend 422302) and 7-AAD, and sorted on a BD FACS Aria II.

After pre-coating cytofunnels with cell staining medium, sorted populations were cytospun on a Thermo Cytospin 4 at 60 g for 5 minutes at room temperature. Additionally, an aliquot of each basophil subpopulation was spotted on a glass plus slide and the cells allowed to settle for 5 minutes. The supernatant was wicked away and the cells fixed with methanol for 5 minutes. All slides were then Wright-Giemsa stained for microscopic imaging. Briefly, slides were stained on an automated stainer for 3 minutes in methanol, 3 minutes in Wright's-Giemsa stain (Beckman Coulter Tru-Color Wright's-Giemsa stain, 7547178), 2 minutes and 30 seconds in stain-buffer combination (50 mL Wright's-Giemsa stain diluted with 90 mL phosphate buffer, pH 6.4), 30 seconds in deionized water, 3 minutes for drying, 1 minute in methanol, 1 minute and 30 seconds in Wright-Giemsa stain, 1 minute in stain-buffer combination, 30 seconds in deionized water, 3 minutes drying. Digital images were taken with a 100x oil objective and Olympus DP22 camera, white balanced and cropped in Adobe Photoshop, and scaled identically. The slides were evaluated by a trained, practicing, board-certified

hematopathologist to classify the cellular phenotypes for each of the "basophil" cell populations. The main morphologic features of basophils are the coarse, basophilic granules which can partially obscure the nucleus and typically two nuclear lobes. Neutrophils have fine, pink granules which do not obscure the nucleus and typically 3-5 nuclear lobes. Both have coarse chromatin and are 10-15 microns.

***Statistical Analysis***

Sidak's multiple comparison tests were conducted to determine significant differences in median intensity of protein expression upon stimulation by anti-IgE or IL-3 in Figure 5 and Figure S3. A p-value resulting from Sidak's multiple comparison tests of < 0.0001 is represented by 4 asterisks (****), 0.0001 to 0.001 by 3 asterisks (***), 0.001 to 0.01 by 2 asterisks (**), 0.01 to 0.05 by one asterisk (*), and if the p value is ≥ 0.05 the difference is determined to not be significant (ns). All analyses and graphical representations were done using a combination of Cytobank, R, Python, and GraphPad Prism.

**SUPPLEMENTAL REFERENCES**

Finck, R., Simonds, E.F., Jager, A., Krishnaswamy, S., Sachs, K., Fantl, W., Pe'er, D., Nolan, G.P., and Bendall, S.C. (2013). Normalization of mass cytometry data with bead standards. Cytometry A *83*, 483-494.

Kotecha, N., Krutzik, P.O., and Irish, J.M. (2010). Web-based analysis and publication of flow cytometry experiments. Curr Protoc Cytom *Chapter 10*, Unit10 17.

Wolf, F.A., Angerer, P., and Theis, F.J. (2018). SCANPY: large-scale single-cell gene expression data analysis. Genome Biol *19*, 15.
